# Water Plays Key Roles in Stabilities of Wild Type and Mutant Transthyretin Complexes

**DOI:** 10.1021/jasms.4c00170

**Published:** 2024-07-26

**Authors:** Carter Lantz, Robert L. Rider, Sangho D. Yun, Arthur Laganowsky, David H. Russell

**Affiliations:** Department of Chemistry, Texas A&M University, College Station, Texas 77843, United States

## Abstract

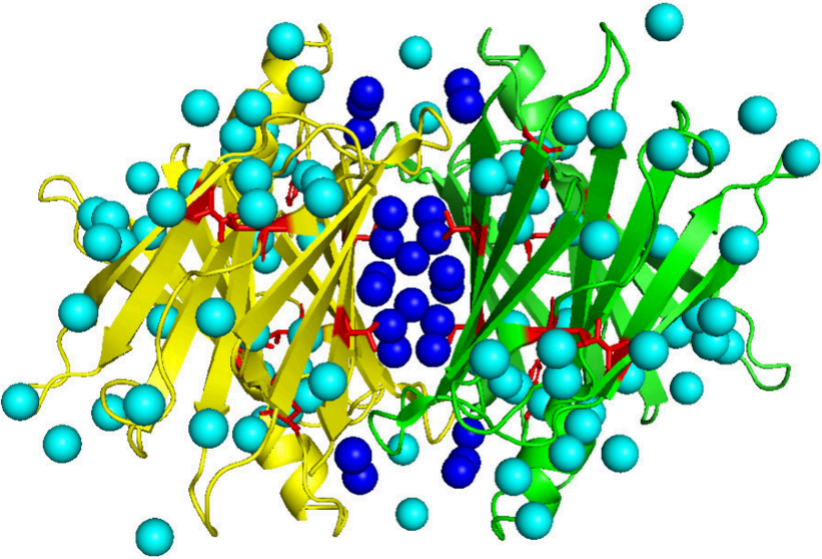

Transthyretin (TTR), a 56 kDa homotetramer that is involved in the transport of thyroxine and retinol, has been linked to amyloidosis through disassembly of tetramers to form monomers, dimers, and trimers that then reassemble into higher order oligomers and/or fibrils. Hybrid TTR (hTTR) tetramers are found in heterozygous individuals that express both wild type TTR (wt-TTR) and mutant TTR (mTTR) forms of the protein, and these states display increased rates of amyloidosis. Here we monitor subunit exchange (SUE) reactions involving homomeric and mixed tetramers using high resolution native mass spectrometry (nMS). Our results show evidence that differences in TTR primary structure alter tetramer stabilities, and hTTR products can form spontaneously by SUE reactions. In addition, we find that solution temperature has strong effects on TTR tetramer stabilities and formation of SUE products. Lower temperatures promote formation of hTTR tetramers containing L55P and V30M subunits, whereas small effects on the formation of hTTR tetramers containing F87A and T119M subunits are observed. We hypothesize that the observed temperature dependent stabilities and subsequent SUE behavior are a result of perturbations to the network of “two kinds of water”: hydrating and structure stabilizing water molecules (Spyrakis et al. J. Med. Chem.2017, 60 ((16)), , 6781−682728475332 10.1021/acs.jmedchem.7b00057; Xu et al. Soft Matter2012, 8, 324–336) that stabilize wt-TTR and mTTR tetramers. The results presented in this work illustrate the utility of high resolution nMS for studies of the structures, stabilities, and dynamics of protein complexes that directly influence SUE reactions.

## Introduction

Transthyretin (TTR) is a 56 kDa tetrameric protein complex that is often investigated due to its unique dynamics and its propensity to cause disease. While TTR has been linked to amyloidosis, both wt-TTR and the T119M mutant protect against amyloid-β toxicity.^1^ L55P and V30M mutations of TTR tend to increase rates of aggregation in organs such as the heart and kidneys,^2−4^ whereas F87A and T119M mutations reduce the amyloidogenic nature of TTR.^5,6^ Heterozygous individuals express both wt-TTR and mTTR subunits, which give rise to hybrid TTR (hTTR) tetramers that have also been linked to amyloidosis. Differences in propensities for mTTR and hTTR complexes to form amyloid aggregates provide evidence that subunit mutations shift tetramer dynamics, and it is possible that further interrogation of these states can provide insight into how disease progresses as well as how inhibitors that suppress amyloidosis can be designed.

**Figure 1:**
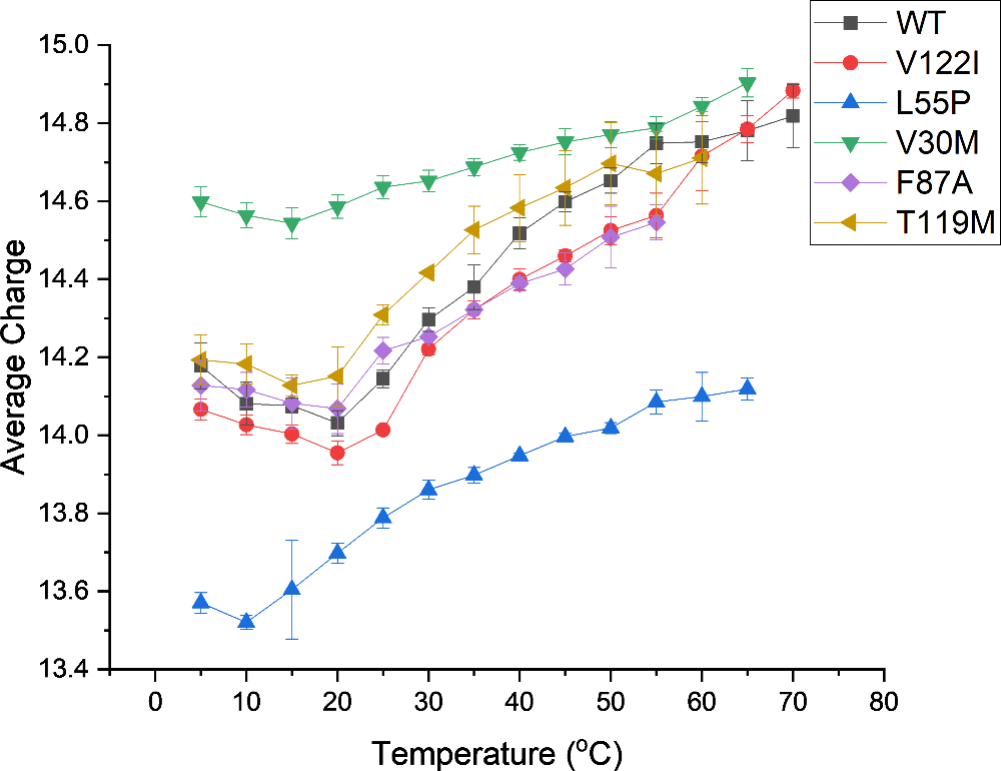
Average charge state (*Z*_avg_) values for TTR tetramers at various temperature values. We interpret the change in *Z*_avg_ as evidence that hydration plays an important role in tetramer stability.

Environmental factors such as post-translational modifications (PTMs),^7,8^ ligand binding,^9−11^ mutations,^12^ and solution conditions (pH, temperature, pressure, presence of osmolytes, and other cofactors)^13−16^ can alter a protein’s complex structure, dynamics, and stability. An important factor that is often overlooked is the role of hydration in protein stability and structure. Xu et al. suggested that there are “two kinds of water” around mutation sites on TTR: (i) water of hydration surrounding stability-bearing mutations that have long residence times; (ii) water adjacent to amyloidogenic mutations that exchange readily with bulk water.^17^ Spyrakis et al. described “cold” and “hot” water molecules in terms of slow and fast exchanges with bulk water, viz., conserved water more slowly exchanges with bulk water.^18^ Others have theorized that changes in hydration can modulate the formation of monomers, dimers, and trimers that have been linked to amyloidosis. An early study showed that pressure changes have strong effects on hydration and packing that are crucial to the amyloidogenesis of TTR mutants.^13^ Banerjee et al. reported that conserved water mediated dynamics of the catalytic H88 residue play key roles in the recognition of thyroxine and retinol binding protein, whereas other conserved water molecules were linked to catalytic and thyroxine binding sites.^19^ These studies specifically link water to structure and dynamics as well as function; however, the details regarding the role of water in these studies were interpreted from crystallographic data and molecular dynamic simulations. Experimental data probing the role of water molecules in TTR stability could provide valuable insight into the dynamics of the TTR tetramer as well as other TTR mutants. Wright et al. recently reported on a novel NMR experiment to determine relative populations of TTR species (tetramer ↔ dimer ↔ monomer) formed by dissociation of a destabilizing TTR mutant (A25T), and their experiments provided both thermodynamic and kinetic parameters including activation energetics for dissociation of the A25T tetramer and enthalpy–entropy compensation (EEC).^20^ Preliminary data from our lab reveals that the charge state of TTR varies with temperature, which we interpret as evidence that changes in the structure of water alter the dynamics of TTR (Figure 1). These studies hint that water molecules could play a key role in stabilizing the TTR tetramer; however, the role of hydration on TTR stability is still largely a mystery.

**Scheme 1:**
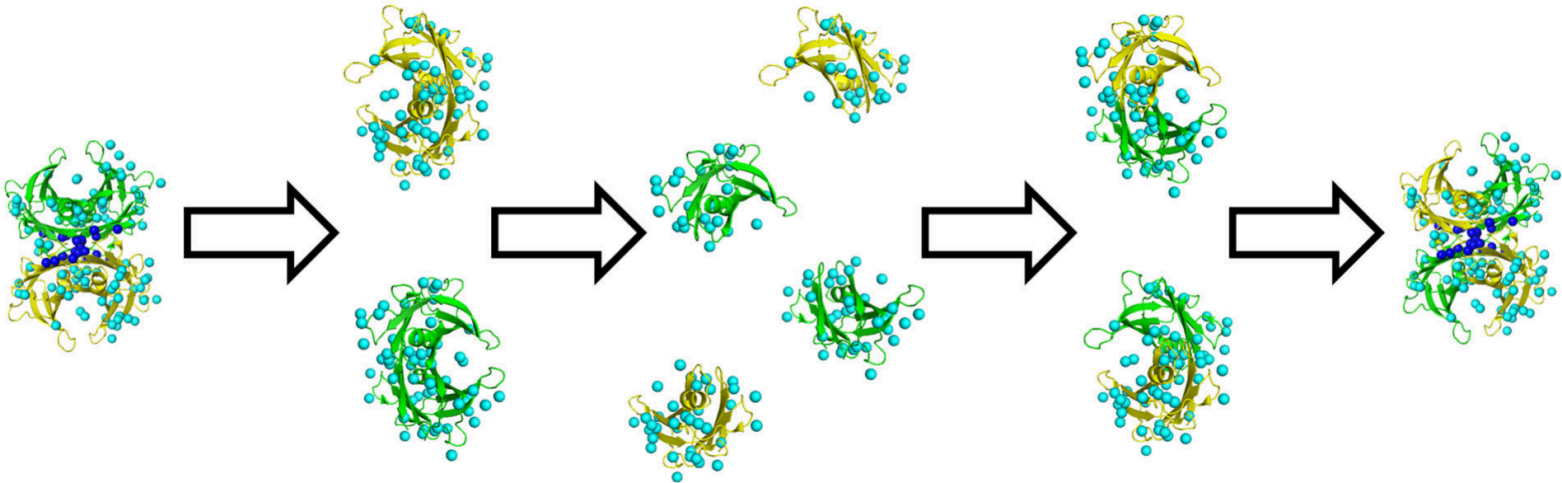
Mechanism of TTR Disassembly and Reassembly Highlighting the Role of Water Molecules in the Process When “cold” water molecules (dark blue spheres) that form the tetramer interface are disrupted, tetramers disassemble into dimers and monomers. During reassembly, interactions between the complex and water molecules re-form; however, it must be noted that the water network of the new tetramer may not be the same as the original water network.

Direct experimental studies of hydration are challenging; thus much of our insight about hydration comes from theory and simulations. Here, we probe the effects of hydration of TTR by investigating the effect(s) of temperature (cold and hot) on subunit exchange (SUE) reactions. The data contained in Figure 1 reveal that the average charges of wt-TTR and TTR mutants change as a function of temperature, presumably owing to changes in the hydration of the TTR complex. Similar changes have been observed in a number of other protein complexes, and such changes have strong effects on the conformations of the complexes as well as ligand binding.^15,21,22^ TTR tetramers disassemble and reassemble to form tetramers containing mixed subunits,^23−26^ and it is possible that cold and hot water molecules present in the crystal structure^27^ could influence SUE by altering the disassembly and reassembly process (Scheme 1). The accepted mechanism for SUE has been extensively debated, and numerous hypotheses have been forwarded. SUE is illustrated using a mechanism introduced by Keetch et al.: disassembly of tetramers to form trimers, dimers, and monomers that reassemble to form TTR tetramers.^23^ Since then, several variants of the original mechanism have been proposed. Foss et al. reported a disassembly mechanism for TTR where tetramers disassemble into dimers and then into monomers.^28^ In 2014, Rappley et al. reported that the disassembled monomers could reassemble into dimers and then tetramers.^24^ This hypothesis is consistent with our published mechanism based on SUE studies of wt-TTR and wt-TTR subunits containing amino acid tags on the N- and C-termini;^26^ however, in the study, a hidden pathway was uncovered showing that dimers of TTR could be exchanged without disassembly into monomers under physiological conditions. While these studies have provided valuable insight into the SUE mechanism, details of tetramer disassembly and reassembly, including the role of hydration, are not yet well understood and should be further explored. In this study, we utilize native mass spectrometry (nMS) to quantify hTTR tetramers formed spontaneously through SUE, without the need for mass tags or isotopic labeling, and our analysis provides information on the role of water in the SUE process.

## Materials and Methods

### Protein Expression

Tetrameric human transthyretin (TTR; Uniprot P02766 residues 21–147) was expressed and purified as previously described^26^ with minor modifications. Residue numbering for the mutants of TTR was denoted based on the post N-terminal signal peptide cleavage. Constructs containing TEV protease cleavable N-terminal 6xHis-MBP-tagged (pET28b) fused to TTR was transformed into BL21 (DE3) *E. coli* cells (New England Biolabs, Ipswich, MA). Colonies were grown in LB at 37 °C until an OD 600 nm value of 0.6–0.8. The cells were induced with 0.5 mM isopropyl β-d-1-thiogalactopyranoside (IPTG) and shaken at 18 °C overnight. Cells were harvested by centrifugation and lysed on ice in the presence of complete protease inhibitor tablet (Roche, Basel, Switzerland) and 5 mM β-mercaptoethanol (β-ME) using an M-110P microfluidizer (Microfluidics, Westwood, MA) at 20 000 psi. Cellular debris was removed by centrifugation at 20000*g* for 25 min. The supernatant was filtered with a 0.45 μm syringe filter before loading onto a HisTrap HP column (Cytiva, Marlborough, MA). The column was equilibrated at ambient temperature with 50 mM TRIS-HCl buffer (pH 7.4), 500 mM NaCl, and 30 mM imidazole (buffer A), and the bound protein was eluted with the same buffer supplemented with 250 mM imidazole. The protein was immediately loaded onto an MBPTrap HP column (Cytiva, Marlborough, MA) equilibrated with buffer A, and the bound protein was eluted with buffer A supplemented with 10 mM d-maltose. The 6xHis-MBP tag was cleaved from TTR by incubation with TEV protease 5 mM β-ME overnight at 4 °C. The tag cleaved proteins were loaded onto a HisTrap HP column equilibrated with buffer A, and the flow-through containing tagless TTR was collected. Tagless TTR was then concentrated using a 50 kDa MWCO centrifugal concentrator (Millipore, Burlington, MA) and subjected to size exclusion chromatography using a Hiload 16/600 Superdex 75 pg column (GE HealthCare, Chicago, IL) equilibrated with 50 mM TRIS-HCl, 150 mM NaCl, and 10% w/v glycerol (pH 7.4) at ambient temperature. Peaks corresponding to the tetrameric TTR were confirmed by mass spectrometry, collected, and concentrated using a 50 kDa MWCO centrifugal concentrator (Millipore, Burlington, MA). Concentrated tagless tetrameric TTR was then diluted with 200 mM ammonium acetate (pH 6.8) to concentrations ranging between 50 and 100 μM, and glycerol was added to reach a final concentration of 20% w/v. Aliquots of TTR (25 μL) were flash frozen with liquid nitrogen and stored at –80 °C.

### SUE Involving Mutant and wt-TTR Homotetramers to Form Hybrid TTR Complexes

To induce SUE between L55P, V30M, T119M, and wt-TTR homotetramers, each solution was buffer exchanged separately with Bio-Spin 6 SEC columns (BioRad, Hercules, CA) into LC–MS grade water at pH 7 (Millipore, Burlington, MA) or 20 mM ammonium acetate at pH 6.8 (Millipore, Burlington, MA). Diethylenetriaminepentaacetic acid (DTPA) with a concentration of 1 mM was added to samples containing coexpressed zinc before being buffer exchanged. Immediately after buffer exchange, an absorbance value was obtained for the mutant and wt-TTR solutions, and the solutions were combined so that the concentration of each TTR species in the solution was 5 μM. Solutions containing wt-TTR and F87A were combined so that the final concentration of F87A TTR was 15 μM and the final concentration of wt-TTR was 5 μM. Once combined, the solutions were incubated at either ambient temperature (∼21 °C) or 35 °C with a MyTemp Mini Digital Incubator (Benchmark Scientific, Sayreville, NJ) and were directly electrosprayed at various time points.

### Native Mass Spectrometry Analysis of SUE Solutions

All experiments were performed on a Thermo UHMR orbitrap mass spectrometer (ThermoFisher Scientific, San Jose, CA). The solutions were loaded into custom pulled borosilicate capillaries pulled with a P-1000 micropipet puller (Sutter Instrument, Novato, CA) and were coated with gold using a Leica EM ACE200 sputter coater (Leica Microsystems, Wetzlar, Germany). To obtain spectra of completely desolvated TTR tetramers, the spray voltage value was 1–2 kV, the DC offset value was 21 V, the desolvation voltage value was 10 V, and the in-source CID value was 50 V. In addition, the trapping gas pressure was set to a value of 4 and the HCD voltage was set to a value of 50–75 V. Data collection was performed at either 25k resolution or 50k resolution. Spectra were collected for 1–5 min, and spectra with sufficiently desolvated tetramer signals were summed. Spectra were deconvoluted using UniDec.^29^ When deconvoluting, the mass range was limited so only tetramers were present in the spectrum, the background was subtracted, the mass was sampled every 1 Da, and the charge state distributions were smoothed. The intensity values from UniDec were utilized to make the histograms.

## Results

**Figure 2:**
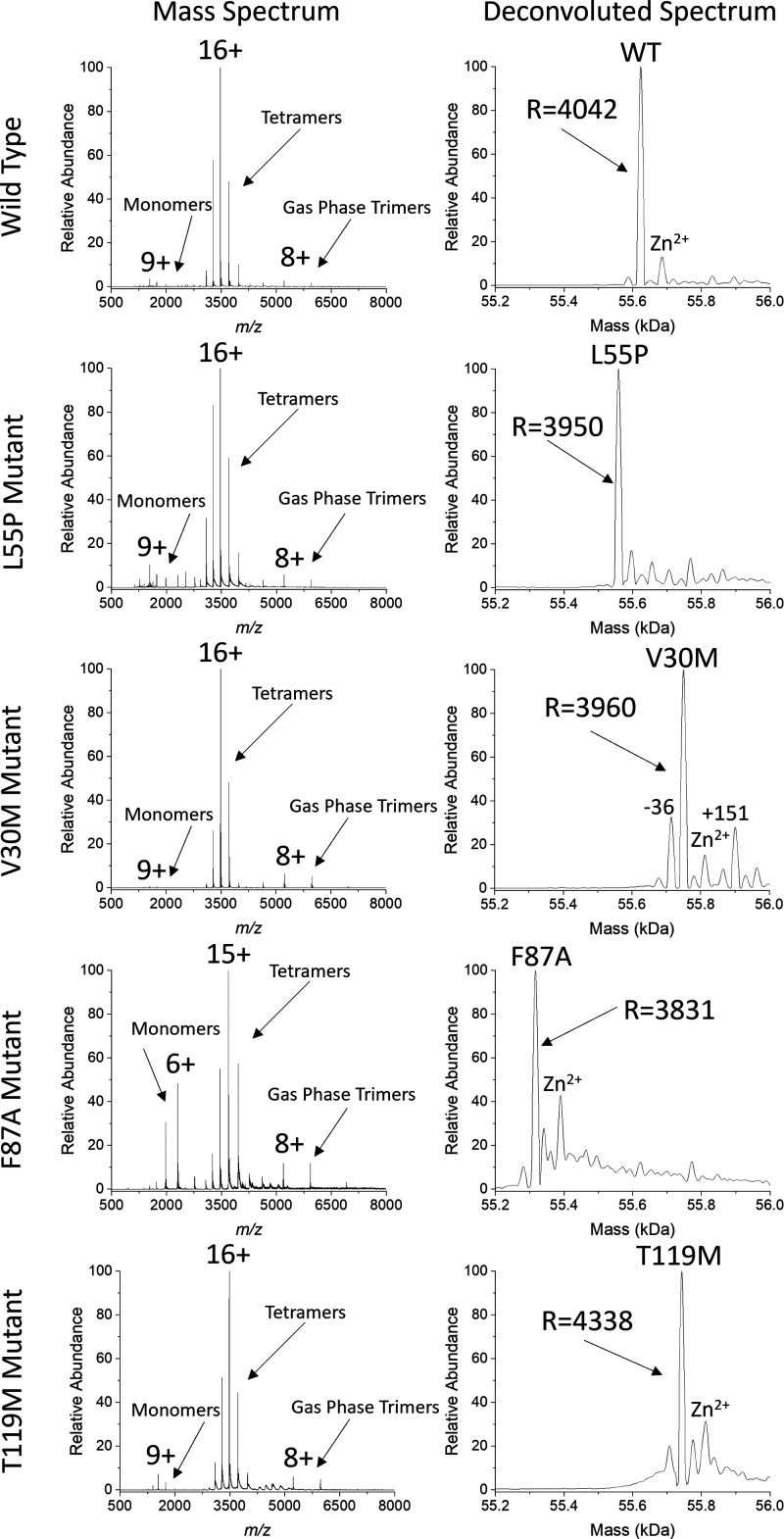
Mass spectra and UniDec deconvoluted spectra for WT, L55P, V30M, F87A, and T119M providing evidence that our method provides enough resolution to separate wtTTR, mTTR, and hTTR states.

To monitor the formation of hTTR tetramers, both homotetramer states and the three hTTR states for each SUE reaction must be sufficiently resolved to be accurately quantified; however, due to the small mass shift induced by TTR mutations, resolving hTTR tetramer states can be difficult. The requirements needed to resolve mTTR and hTTR tetramers relative to wt-TTR are illustrated in Table 1. Previous studies have utilized mass tags^26,30^ or isotopic labeling^23,25^ to quantify hTTR tetramers and provide information on the mechanism and rate of SUE, although adding modifications to TTR often induces mass shifts that overlap with adjacent species^23,25^ or even alters complex stability.^31^ For example, Keetch et al. reported that the 16+ charge state (CS) of [^13^C–^15^N] labeled wt-TTR overlapped with the 15+ charge state of [^12^C–^14^N]-TTR, making quantification of these tetramer states difficult.^23^ In another study, it was reported that dual-FLAG tag TTR tetramers were more stable (i.e., less tetramer dissociation) than wt-TTR tetramers, indicating that the dual-FLAG tag modification may alter SUE results.^31^ Our nMS results show evidence that, under our solution and instrument conditions, tetramers and low abundance monomers and trimers are detected (Figure 2). Monomer and trimer signals in the spectra are from gas phase activation needed to fully desolvate the tetramers. Abundant solution phase monomer signals are present in the F87A spectrum because of its instability in water. Signals corresponding to TTR homotetramers have a resolving power of ∼4000, and adduct signals are not abundant in the spectra. This method allows for accurate quantification of hTTR tetramers and monitoring of SUE reactions between wt-TTR and various mTTR homotetramers. In this study, SUE reactions were induced by dissolving intact homotetramers in water solutions unless otherwise stated, and nMS was performed on the solutions at various time points. Concentration of TTR in solution was based on the absorbance of all states in solution, which may account for some variability in abundance at *t* = 0.

**Table 1:** Requirements for Resolving and Quantifying hTTR Tetramers Containing mTTR and wt-TTR Subunits

mutant	average monomer mass (Da)	average tetramer mass (Da)	Δmass of tetramer from wt-TTR (Da)	Δmass of monomer from wt-TTR (Da)	Δ*m*/*z* shift for each hTTR state (16+ CS)	resolving power needed to separate hTTR tetramers
WT	13906	55622	NA	NA	NA	NA
L55P	13889	55558	–64.20	–16.05	–1.00	3462
V30M	13938	55750	128.24	32.06	2.00	1739
F87A	13829	55318	–304.40	–76.10	–4.76	727
T119M	13936	55742	120.32	30.08	1.88	1853

### Stability of Mutant TTR Homotetramers Dictates Subunit Exchange

**Figure 3:**
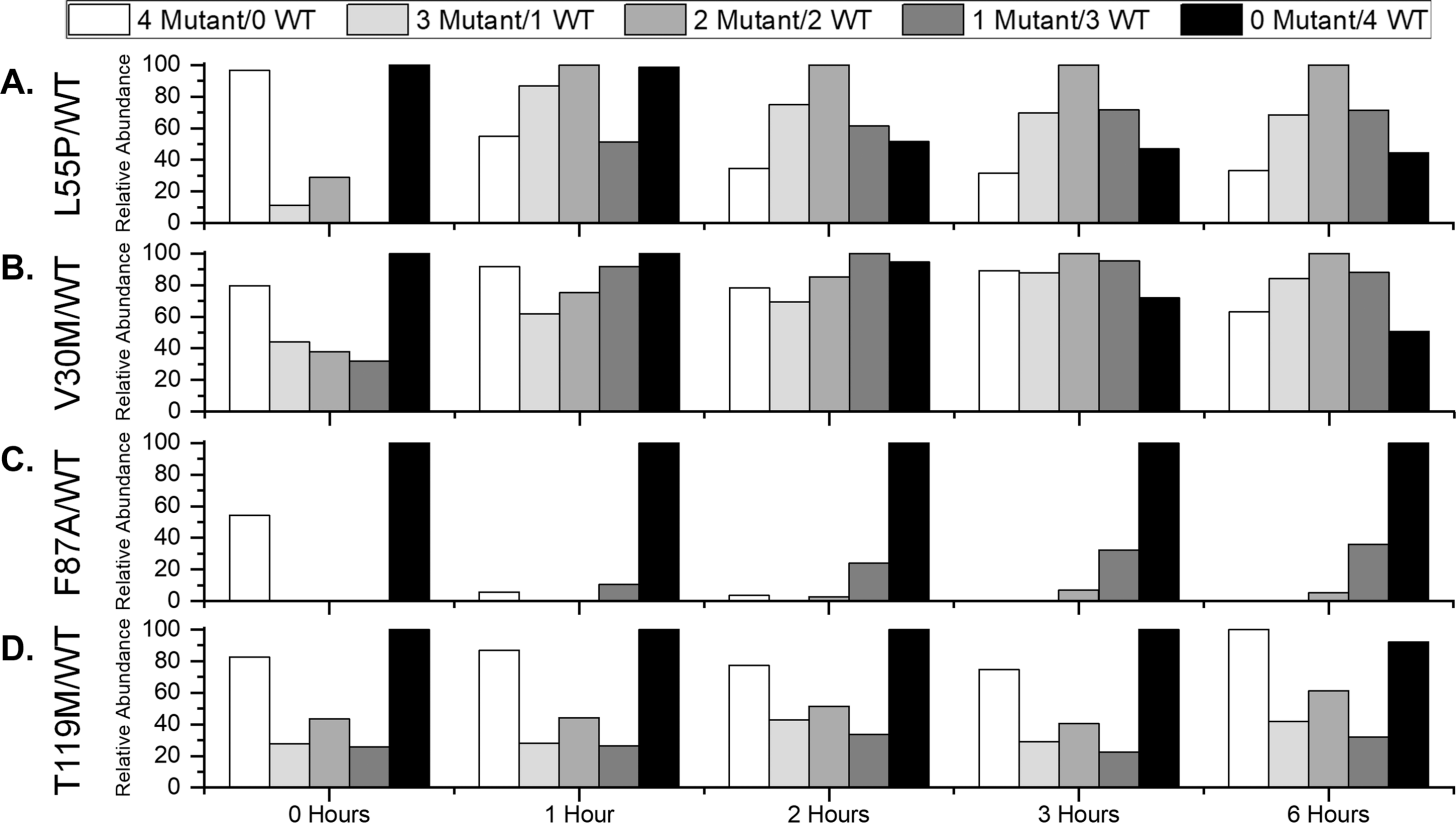
Histograms of relative abundance values for wt-TTR, mTTR, and hTTR tetramers present in 21 °C solutions initially containing wt-TTR and (A) L55P, (B) V30M, (C) F87A, and (D) T119M homotetramers. The relative abundance values provide evidence that SUE occurs spontaneously in water and that stability of mutant tetramers dictates the SUE process.

The L55P mutation has been linked to increased aggregation of TTR, and hTTR tetramers with L55P subunits are presumed to be disease states that accelerate amyloidosis. To characterize the formation of hTTR complexes containing L55P subunits, a solution containing wt-TTR and L55P homotetramers was incubated at equimolar concentration at ambient temperature (∼21 °C) before direct electrospraying of the solution at various time points (Figure 3A; Figure S1). The histograms show evidence that, as time proceeds, signals corresponding to hTTR complexes increase in abundance, providing evidence that SUE occurs spontaneously in water. Furthermore, L55P homotetramers decrease in abundance at earlier time points compared to wt-TTR homotetramers, providing evidence that L55P homotetramers are less stable than wt-TTR homotetramers under these conditions. At earlier time points, hTTR tetramers that contain three L55P subunits are in greater abundance compared to hTTR tetramers with one L55P subunit, which provides evidence that disassembly of L55P homotetramers promotes formation of hTTR tetramers. Furthermore, we interpret increased abundances of hTTR tetramers at later time points to mean that hTTR tetramers remain stable in water over time. At 6 h, the distribution of tetramers was relatively Gaussian, providing evidence the SUE reaction was nearly at equilibrium at this time point. Monitoring SUE between L55P and wt-TTR homotetramers with nMS provides evidence that L55P homotetramers are less stable than wt-TTR homotetramers and that hTTR states form spontaneously within 6 h.

The V30M mutation has also been reported to be amyloidogenic. To characterize formation of hTTR complexes containing V30M subunits, wt-TTR and V30M homotetramers were incubated at equimolar concentration at ambient temperature and the solutions were electrosprayed at various time points (Figure 3B; Figure S2). Signals corresponding to hTTR tetramers increase in abundance over time, providing evidence that SUE between these homotetramers occurs spontaneously in water. wt-TTR homotetramers decrease in abundance more readily compared to V30M homotetramers, providing evidence that V30M homotetramers are more stable in water compared to wt-TTR homotetramers. In addition, the histograms show evidence that hTTR tetramers with one V30M subunit increase in abundance more readily than hTTR tetramers with three V30M subunits, providing evidence that, at early time points, disassembly of wt-TTR homotetramers drives SUE. We interpret the abundance values of hTTR tetramers at later time points to mean that hTTR tetramers remain stable in water over time. Similar to SUE between L55P and wt-TTR, a Gaussian distribution of tetramers was apparent at 6 h, providing evidence the SUE reaction was nearly at equilibrium at this time point. Monitoring SUE between V30M and wt-TTR homotetramers with nMS provides evidence that V30M homotetramers are more stable than wt-TTR homotetramers but that hTTR tetramers still form spontaneously within 6 h.

Stabilities of TTR complexes containing L55P and V30M subunits are such that a Gaussian distribution of tetramers containing mTTR and wt-TTR subunits is apparent within 6 h; however, it has been reported that other TTR mutations more readily alter the stabilities of TTR complexes. The F87A mutation reduces the stability of the tetramer compared to wt-TTR, and due to the reduced number of contacts with adjacent subunits, it does not aggregate as readily.^5^ To probe SUE behavior between F87A and wt-TTR complexes, wt-TTR and F87A homotetramers were incubated at a 1:3 molar ratio at ambient temperature and electrosprayed at various time points (Figure 3C; Figure S3). At time 0, signals corresponding to F87A homotetramers and wt-TTR homotetramers are present in the spectrum. At 1 h, F87A homotetramers decreased in abundance, providing evidence that F87A homotetramers are less stable in water compared to wt-TTR homotetramers. As time progressed, a signal corresponding to hTTR tetramers with one F87A subunit and a low abundance signal corresponding to tetramers containing two F87A subunits did appear, but a signal corresponding to tetramers containing three F87A subunits did not appear. We interpret the low abundance of tetramer signals containing F87A subunits to mean that the addition of F87A subunits decreases complex stability. Decreased stability of TTR complexes with F87A subunits prevents formation of hTTR tetramers and progression of SUE.

The T119M mutation has been reported to stabilize TTR tetramers.^32^ To probe the formation of hTTR complexes containing T119M subunits, an equimolar amount of wt-TTR and T119M homotetramers was incubated in a solution of water at ambient temperature and analyzed at various time points (Figure 3D; Figure S4). The histograms show evidence that hTTR tetramers do not increase in abundance as readily compared to hTTR tetramers containing L55P or V30M subunits. Even at 6 h, hTTR tetramer signals were not more abundant compared to their abundance at 0 h. Furthermore, the histograms show evidence that both T119M homotetramers and wt-TTR homotetramers are present in solution after 6 h, demonstrating that hTTR tetramers do not form because T119M homotetramers do not dissociate readily. Although many homotetramers stay intact, we do observe that wt-TTR homotetramers decreased in abundance compared to T119M homotetramers, providing evidence that T119M homotetramers are more stable compared to wt-TTR homotetramers. Analysis of SUE between T119M and wt-TTR homotetramers provides evidence that T119M homotetramers are more stable than wt-TTR homotetramers and the stability provided by T119M prevents formation of hTTR tetramers.

Shirzadeh et al. previously reported that higher salt concentrations slow SUE.^26^ To determine how salt concentration affects SUE progression, L55P and wt-TTR homotetramers were added to a solution at ambient temperature containing 20 mM ammonium acetate, and tetramer abundances were monitored over 6 h (Figure S5). The results show evidence that hTTR tetramers formed spontaneously in this solution but were not formed as readily compared to the formation of hTTR tetramers in water, which provides evidence that SUE is hindered in higher salt concentrations. It was also observed that sodium adducts were present when electrospraying in 20 mM ammonium acetate, providing some ambiguity at early time points. Similar observations were recorded when V30M homotetramers were incubated with wt-TTR homotetramers in 20 mM ammonium acetate. SUE in ammonium acetate shows evidence that hTTR tetramers did form spontaneously but did not form as readily in high salt conditions (Figure S6). We interpret decreases in abundance of hTTR tetramers when ammonium acetate is inserted into the solution to mean that ionic strength is an important factor for tetramer stability and SUE.

### Temperature Dependence of SUE

**Figure 4:**
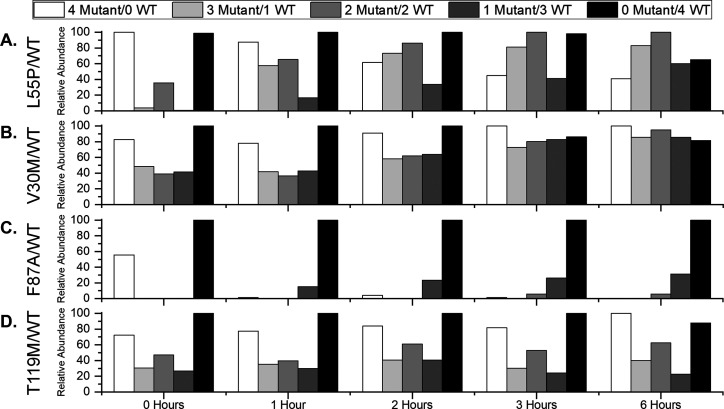
Histograms of relative abundance values for wt-TTR, mTTR, and hTTR tetramers present in 35 °C solutions initially containing wt-TTR and (A) L55P, (B) V30M, (C) F87A, and (D) T119M homotetramers. The relative abundance values show evidence that, in many cases, hTTR formation is hindered at 35 °C, which we interpret to mean that hydration plays a role in SUE.

Previous work by our lab and others illustrates that changes in solution temperature can alter the conformational dynamics of proteins and protein complexes by altering the network of water molecules surrounding them.^15,33^ Changes in hydration can affect key characteristics of those proteins such as enzyme activity and ligand binding affinity, among others. To determine how temperature affects SUE behavior, a solution containing wt-TTR and L55P homotetramers was incubated at 35 °C and monitored at various time points (Figure 4A; Figure S7). The histograms show evidence that SUE proceeds in a similar manner at 35 °C compared to ambient temperature and that L55P is still less stable than wt-TTR; however, abundances of hTTR states do not increase as readily at 35 °C, providing evidence that SUE is hindered at higher temperature. This observation is especially evident at 2 h, which reveals a near Gaussian distribution of tetramers at ambient temperature (Figure 3A) but not at 35 °C (Figure 4A). A similar trend was observed for V30M. Monitoring SUE in a solution containing wt-TTR and V30M homotetramers incubated at 35 °C provides evidence that formation of hTTR tetramers proceeds in a similar manner compared to SUE at ambient temperature, but formation of hTTR tetramers is hindered (Figure 4B; Figure S8). This observation is especially evident at 6 h, which reveals a Gaussian distribution for TTR states at ambient temperature (Figure 3B) but not at 35 °C (Figure 4B). We interpret the observation that formation of hTTR complexes containing L55P and V30M subunits is hindered by an increase in temperature to mean that SUE involving these mutants is sensitive to changes in hydration.

Analysis of the mole fraction of each species in solutions containing wt-TTR tetramers and L55P or V30M tetramers further demonstrates the temperature dependence of hTTR formation. The plot of L55P and wtTTR at 21 °C reveals that the mole fraction of wtTTR and L55P homotetramers decreases in abundance over time and that the mole fraction of hTTR tetramers increases in abundance over time (Figure S9A). When the temperature is increased to 35 °C, the mole fraction of wt-TTR and L55P homotetramers decreases at later time points and the mole fraction of hTTR tetramers increases at later time points compared to 21 °C (Figure S9B). We interpret the decrease in mole fraction of homotetramers at later time points at 35 °C as evidence that wt-TTR and L55P TTR stability at 35 °C is greater than at 21 °C. Similar results are provided for SUE reactions between wt-TTR and V30M homotetramers. At 21 °C, the mole fraction of wt-TTR and V30M homotetramers decreases in abundance over time and the mole fraction of hTTR tetramers increases in abundance over time (Figure S9C). When the temperature of the solution is increased to 35 °C, the mole fraction of wt-TTR and V30M homotetramers decreases at later time points and the mole fraction of hTTR tetramers increases at later time points compared to 21 °C (Figure S9D). We interpret the decrease in mole fraction of homotetramers at later time points at 35 °C as evidence that wt-TTR and V30M stability at 35 °C is greater than at 21 °C. The variation of species mole fraction in solution as a function of temperature further demonstrates that hydration plays a role in SUE reactions involving wt-TTR, L55P, and V30M tetramers.

Temperature appears to be a determining factor for SUE of L55P and V30M with wt-TTR; however, it is not for SUE with F87A and T119M mutants. A solution containing F87A and wt-TTR homotetramers at a 3:1 molar ratio was incubated at 35 °C and electrosprayed at various time points (Figure 4C; Figure S10). The histograms show evidence that, at 1 h, the signal corresponding to F87A homotetramers was low in abundance. Over time, low abundance signals corresponding to hTTR tetramers containing one F87A subunit or two F87A subunits appeared. Abundance values for tetramers present in solution at 35 °C (Figure 4C) do not change relative to tetramers present in solution at ambient temperature (Figure 3C), providing evidence that temperature does not significantly affect SUE behavior involving F87A subunits. Effects of temperature on SUE reactions involving T119M subunits were also investigated. A solution containing T119M and wt-TTR homotetramers at a 1:1 molar ratio was incubated at 35 °C and electrosprayed at various time points (Figure 4D; Figure S11). The histograms show evidence that abundance values for T119M and wt-TTR homotetramers and hTTR tetramers did not change over 6 h, which is similar to the results at ambient temperature (Figure 3D). The fact that similar results were collected at 35 °C compared to ambient temperature provides evidence that temperature does not affect formation of hTTR complexes containing T119M subunits. We interpret the inability for changes in temperature to alter abundances of hTTR complexes containing F87A or T119M subunits to mean that SUE involving these mutants is not affected by changes in hydration.

Analysis of the mole fraction of each species in solutions of wt-TTR and F87A or T119M further demonstrates that temperature does not affect formation of hTTR tetramers. The plot of F87A and wt-TTR at 21 °C reveals that the mole fraction of wt-TTR does not change, the mole fraction of F87A homotetramers decreases, and the mole fraction of some hTTR tetramers increases slightly over time (Figure S9E). When the temperature of the solution is increased to 35 °C, the mole fraction plot does not shift compared to the mole fraction plot at 21 °C (Figure S9F). We interpret the similar results at 21 and 35 °C as evidence that SUE involving F87A is not affected by changes in solution temperature under our conditions. Our results also provide evidence that SUE with T119M is not affected by solution temperature. The plot of T119M with wt-TTR at 21 °C reveals that the mole fractions of all species do not change over the course of 6 h (Figure S9G). When the solution of the temperature is increased to 35 °C, the mole fraction of the species in solution does not change (Figure S9H). We interpret the similar results at 21 and 35 °C as evidence that SUE involving T119M is not affected by changes in temperature under our conditions. The observation that temperature does not affect formation of hTTR tetramers containing F87A and T119M subunits further demonstrates that changes in hydration do not affect SUE reactions involving these mutants at the given time points.

### Insights into the SUE Mechanism of TTR

**Scheme 2:**
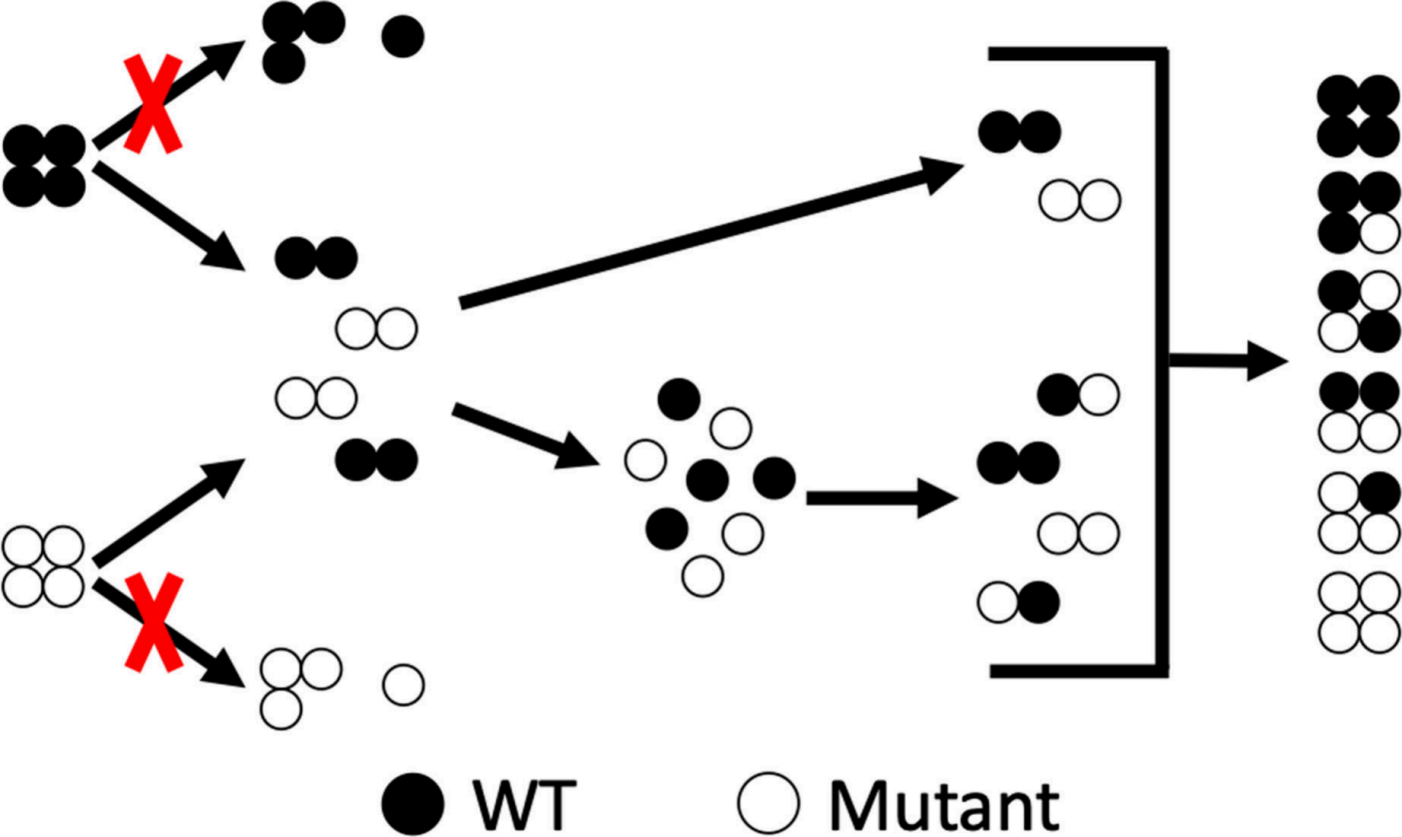
Proposed SUE Mechanism between wt-TTR and mTTR Homotetramers Based on Abundances of TTR Species in Solution

Relative abundances of TTR species in solution shed light on the SUE mechanism of TTR. Mass spectra of solutions initially containing L55P and wt-TTR homotetramers or V30M and wt-TTR homotetramers provide evidence for abundant monomer products and lower abundance dimer products at each time point (Figure S12). Solutions initially containing F87A and wt-TTR homotetramers contained abundant monomer products at each time point (Figure S13). Solutions initially containing T119M and wt-TTR homotetramers provide evidence for low abundance monomer products at each time point (Figure S14). None of the spectra provides evidence for solution phase trimers. We interpret the presence of monomer and dimer products in the mass spectra to mean that SUE begins by disassembly of homotetramers into dimer products that then further disassemble into monomer products (Scheme 2). The high abundance of monomer products compared to dimer products provides evidence that disassembly of tetramers into dimers is the limiting step in the reaction. The mass spectra also provide evidence that hybrid dimers are present (Figure S15). The presence of hybrid dimers suggests that assembly of hTTR tetramers occurs when monomers reassemble to form dimers that then reassemble to form tetramers (Scheme 2). It is worth noting that low abundance trimers are observed in the high *m*/*z* region of the spectra, but due to their low charge, they can be assigned as gas phase trimers that are released during desolvation of TTR tetramers.^34^ We interpret the abundance of monomers and dimers in the spectra to mean that the dominant mechanism for SUE under these conditions proceeds by disassembly of TTR tetramers into dimers and then monomers; the free monomers in solution then reassemble into dimers that then form tetramers.

The abundance of hTTR tetramer species in solution provides evidence that SUE can proceed by more than one mechanism. Figure 3A reveals that, at 0 h, the signal corresponding to tetramers containing two L55P subunits is more abundant than signals corresponding to tetramers containing one L55P subunit or three L55P subunits but is less abundant than signals representing both homotetramers. This observation is also recorded for the 0-h time point and 1-h time point in Figure 4A. This is also the case for solutions containing T119M and wt-TTR homotetramers. In Figure 3D and Figure 4D, signals corresponding to tetramers containing two T119M subunits are more abundant than signals corresponding to tetramers containing one T119M subunit or three T119M subunits but are less abundant than signals for both homotetramers. We interpret the observation that hTTR tetramers containing two mutant subunits are relatively abundant at early SUE time points to mean that SUE can occur by exchange of dimers without disassembly into monomers (Scheme 2). This does not seem to be the case for SUE between V30M and wt-TTR homotetramers and F87A and wt-TTR homotetramers. In Figure 3B,C and Figure 4B,C, hTTR tetramers containing two mutant and two wt-TTR subunits are not significantly more abundant than hTTR tetramers containing three mutant subunits or one mutant subunit. We interpret the abundance of tetramers containing two wt-TTR and two mTTR subunits to mean that, for some mutants, SUE can occur by exchange of dimer products between tetramers.

## Discussion

The experiments conducted in this study provide key information on TTR tetramer stabilities and reveal that those stabilities dictate how SUE proceeds. Both L55P and V30M readily exchange subunits with wt-TTR at ambient temperature and form a Gaussian distribution of tetramers with wt-TTR subunits within 6 h; however, homotetramer stabilities determine which hTTR tetramers are formed at early time points. Homotetramers containing L55P subunits are less stable than homotetramers containing wt-TTR subunits, so disassembly of L55P homotetramers promotes hTTR formation at early time points as is evident by the increased relative abundance of tetramers with three L55P subunits (Figure 3A). On the other hand, V30M homotetramers are slightly more stable than wt-TTR homotetramers, so disassembly of wt-TTR homotetramers promotes hTTR formation at early time points as is evident by the increased relative abundance of tetramers with one V30M subunit (Figure 3B). Previous NMR data provides evidence that the L55P and V30M mutations slightly perturb the β sheet regions of TTR monomers;^35^ however, the overall structure of TTR does not change significantly. Since quaternary structure differences are small, it is possible that observed differences in mTTR and hTTR tetramer stabilities are indicative of a shift in the network of water molecules surrounding the complex in solution phase. A previous TTR study involving mutation of H88 provides evidence that alteration of the TTR water network destabilizes TTR tetramer structure.^36^ We hypothesize that L55P and V30M mutations could also shift the network of water molecules surrounding TTR, which would alter tetramer stability and formation of hTTR complexes during SUE.

Other mutations prevent formation of hTTR tetramers. Tetramers containing subunits with the F87A mutation are unstable in water, which is illustrated by low abundance values of F87A homotetramers at 1 h and only a moderate increase in hTTR tetramer abundance over 6 h (Figure 3C). Previous studies provide evidence that F87 is involved in formation of the transthyretin dimer interface by a packing mechanism with the adjacent subunit^27^ and that displacement of the F87 side chain from its binding pocket promotes tetramer disassembly.^37^ Removal of the phenyl group from the pocket could disrupt the structure of TTR and destabilize TTR tetramers. Destabilization would prevent formation of F87A homotetramers and hTTR tetramers containing F87A subunits. Conversely, the T119M mutation prevents formation of hTTR tetramers due to increased stability of tetramers containing subunits with the mutation. The histograms show evidence that wt-TTR homotetramers decrease in abundance relative to T119M homotetramers over 6 h at ambient temperature, which we interpret to mean that T119M homotetramers are more stable than wt-TTR homotetramers (Figure 3D). A previous NMR study discussing structural shifts of various TTR mutants provides evidence that T119M perturbs the structure of a loop region near the C-terminus, which differs from the region perturbed by L55P or V30M.^35^ Another study provides evidence that water molecules cluster around that same loop region near the C-terminus of TTR.^17^ From the observations made in these studies, it may be concluded that T119M shifts the structure of TTR subunits and stabilizes tetramer formation. Stabilization of T119M tetramers would prevent disassembly and inhibit their ability to undergo SUE reactions with wt-TTR. Stabilities of tetramers containing F87A and T119M subunits are such that hTTR complexes do not form through SUE, and their altered stability could be the reason these mutants do not readily form amyloid oligomers and fibrils.

One observation we find enlightening is that changes in solution temperature alter the formation of some hTTR tetramers, which we interpret as evidence that water in solution phase plays a role in stabilizing TTR. In the case of SUE between wt-TTR and L55P, relative abundance values for hTTR tetramers are greater and relative abundance values for homotetramers are lesser when the solution temperature is lower, providing evidence that hTTR formation is promoted at lower temperatures (Figure S16A). The same trend is observed for tetramers formed during SUE between V30M and wt-TTR (Figure S16B). Changes in solution temperature often induce changes in protein dynamics, which is frequently attributed to changes in water structure.^33,38^ Previous studies by Spyrakis et al. and Banerjee et al. detail the role of “cold” water molecules (i.e., less dynamic water molecules) in stabilizing protein structure.^18,39^ It is known that the dimer interface of TTR is formed by hydrophobic contacts between the AB and GH loops and a network of these cold water molecules that form hydrogen bonds with the backbone of TTR and other residues.^39,40^ Previous research has revealed that temperature changes alter the network of water molecules around TTR and that cold temperatures increase hydration of hydrophobic regions.^41−44^ These studies provide evidence that water molecules are important for TTR stability, and changes in solution temperature could disrupt the network of cold water molecules forming the dimer interface of TTR. Alterations of water structure would compromise TTR structure and promote disassembly and subsequent SUE. The observation that temperature alters abundances of TTR tetramers during SUE illustrates that water molecules are an important part of TTR structure and perturbation of the water network destabilizes tetramers and facilitates SUE.

Temperature changes do not affect SUE reactions involving F87A and T119M subunits. In the case of SUE between F87A and wt-TTR homotetramers, only small differences in tetramer abundance values are apparent when the solution temperature is changed (Figure S16C). We interpret the observation that formation of hTTR tetramers is not affected by temperature to mean that the network of water molecules stabilizing TTR tetramers is already disrupted by the F87A mutation. Disruption of water structure by removing the phenyl group would diminish the effect temperature would have on the complex stability. Likewise, small differences in abundance values are apparent at different temperatures for SUE between T119M and wt-TTR homotetramers (Figure S16D); however, this is due to increased stability of T119M homotetramers. T119M recruits water molecules to the dimer interface of the tetramer.^17^ Those water molecules could provide additional stability to TTR tetramers by resisting changes in water structure. A more stable structure would prevent disassembly of T119M homotetramers and progression of SUE.

Mass spectra collected during SUE show evidence that SUE proceeds by a disassembly/reassembly mechanism of tetramer species. The primary mechanism by which SUE occurs involves disassembly of tetramers into dimers and then monomers that then reassemble into dimers and then tetramers. This mechanism closely resembles the mechanism proposed by Rappley et al.^24^ and Shirzadeh et al.^26^ that both describe a process of disassembly and reassembly of TTR tetramers. The inability for TTR tetramers to disassemble or reassemble inhibits SUE between tetramers. T119M prevents homotetramer disassembly and thus does not produce free monomers needed for SUE to progress. Conversely, F87A prevents reassembly of TTR tetramers once they are in monomer form and thus does not form tetramers to complete the SUE process. The mass spectra also provide evidence that solution phase trimer products are not present in solution. We interpret the absence of trimer products in our mass spectra to mean that, when incubated in water, tetramers do not disassemble into trimer and monomer products. Even though we do not observe trimer formation in our study, it is possible that, under other conditions, these states may form.

The histograms show evidence that a less prominent mechanism involves exchange of dimer species without disassembly into monomers. Figure 3A,D shows evidence that, at early time points, an abundant signal corresponding to tetramers containing two mutant and two wt-TTR subunits is present providing evidence dimer exchange can occur when L55P or T119M exchanges with wt-TTR. Keetch et al. hypothesized that dimer exchange was part of the SUE pathway and that it could occur by assembly of two free dimer products or by formation of a hexamer product.^23^ In our mass spectra, we do not find evidence of a hexamer product, so we conclude that free dimers in solution reassemble into tetramer products without hexamer intermediates. This mechanism also agrees with our previous work revealing a hidden mechanism involving dimer exchange.^26^ Interestingly, dimer exchange seems to only contribute to SUE for certain mutants. Figure 3B,C does not show evidence of an abundant signal corresponding to tetramers containing two mutant and two wt-TTR subunits, providing evidence that V30M and F87A do not exchange dimers with wt-TTR. The reason dimer exchange is present for some mutants but not for other mutants is not currently known and warrants further investigation.

## Conclusion

Accurate characterization of protein complexes with nMS can reveal relevant information on their compositional entropy (i.e., the driving force that facilities heterogeneity of protein complexes) and provide insight into protein function, structural dynamics, and disease mechanisms. Understanding how TTR compositional entropy develops may provide insight into how TTR amyloidosis progresses. This study details an nMS method that can be utilized to monitor SUE of TTR subunits between wt-TTR and mTTR homotetramers without the use of mass tags or isotopic labeling. Our results show evidence that the primary structure of TTR shifts SUE behavior presumably by altering homotetramer stability. Mutant homotetramers of L55P and V30M readily exchange subunits with wt-TTR homotetramers within 6 h; however, our results provide evidence that disassembly of L55P homotetramers promotes formation of hTTR tetramers with wt-TTR while disassembly of wt-TTR homotetramers promotes formation of hTTR tetramers with V30M. Mutations with more drastic stability alterations (e.g., F87A and T119M) do not readily form hTTR tetramers with wt-TTR, presumably due to alterations in complex hydration. Our results show evidence that the SUE mechanism proceeds by disassembly of tetramers into dimer products that then disassemble into monomer products; free monomers then reassemble into dimers that then form tetramer products. This mechanism resembles the one proposed by Rappley et al. and previously by our lab.^24,26^ In addition, the mass spectra collected hint that dimer exchange can occur for some mutants (e.g., L55P and T119M), which is a mechanism that has been reported by Keetch et al. and previously by our lab.^23,26^ However, other mutants studied (i.e., V30M and F87A) do not exchange dimers, further illustrating that the primary structure of TTR mutants affects SUE behavior. The results presented provide information on the stability of TTR tetramer states and shed light on the mechanism that leads to the formation of hTTR tetramers.

Our results also provide evidence that temperature changes alter TTR stability and formation of hTTR states. Specifically, mass spectra collected show evidence that hTTR states are more readily formed at ambient temperature compared to 35 °C in solutions containing V30M and L55P subunits, providing evidence that TTR complexes are destabilized at lower temperature. Previous studies have hypothesized that “cold” water molecules can be integrated into protein structures and stabilize protein/ligand interactions as well as protein/protein interactions.^18^ TTR crystal structures show evidence that numerous water molecules span the hydrophobic dimer interface of TTR tetramers, which may help stabilize tetramer formation; one study in particular determined that there are eight water molecules that are integral in forming the dimer interface.^39^ We interpret the observation that decreased temperatures promote formation of hTTR tetramers to mean that, at lower temperatures, the network of water molecules stabilizing TTR homotetramers is disrupted. Disruption of water structure promotes disassembly of tetramers and begins SUE. Temperature dependence of hTTR formation highlights the role water molecules play in the stabilization of TTR tetramer structure and provides a rationale for TTR disassembly and progression of SUE. The observation that disruption of water molecule structure can promote hTTR formation may indicate that environmental factors known to affect hydration (i.e., temperature, osmolytes, pH, chemical denaturants) could play a role in progression of TTR diseases including amyloidosis.

## Supporting Information Available

Raw mass spectra collected during SUE experiments and UniDec deconvoluted plots of TTR tetramers (PDF)
