# Supplementary material for: Water Plays Key Roles in Stabilities of Wild Type and Mutant Transthyretin Complexes
Source: J Am Soc Mass Spectrom. 2024 Jul 26;35(8):1854–64. doi: 10.1021/jasms.4c00170 (PMC11311534; doi:10.1021/jasms.4c00170)
Supplement: Supplementary file 1 — js4c00170_si_001.pdf [file js4c00170_si_001.pdf]

Supporting information

Water Plays Key Roles in Stabilities of Wild Type and Mutant Transthyretin Complexes

Carter Lantz, Robert L. Rider, Sangho D. Yun, Arthur Laganowsky, David H. Russell*

Department of Chemistry, Texas A&M University, College Station, TX 77843

*Corresponding author; Email: russell@chem.tamu.edu

Table of Contents **Page**

Figure	Page
Figure S1	S2
Figure S2	S2
Figure S3	S2
Figure S4	S2
Figure S5	S3
Figure S6	S3
Figure S7	S3
Figure S8	S3
Figure S9	S4
Figure S10	S5
Figure S11	S5
Figure S12	S5
Figure S13	S6
Figure S14	S6
Figure S15	S6
Figure S16	S7

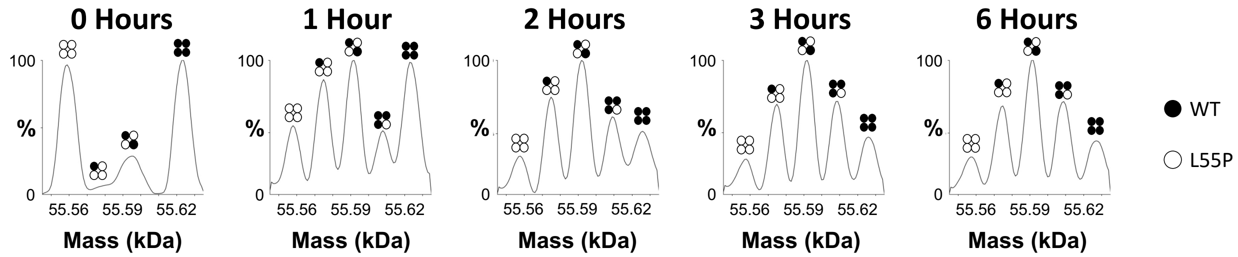

Figure S1: UniDec deconvoluted plots showing SUE between WT and L55P homotetramers in water at ambient temperature (21 °C). Notice that hTTR tetramers increase in abundance over time.

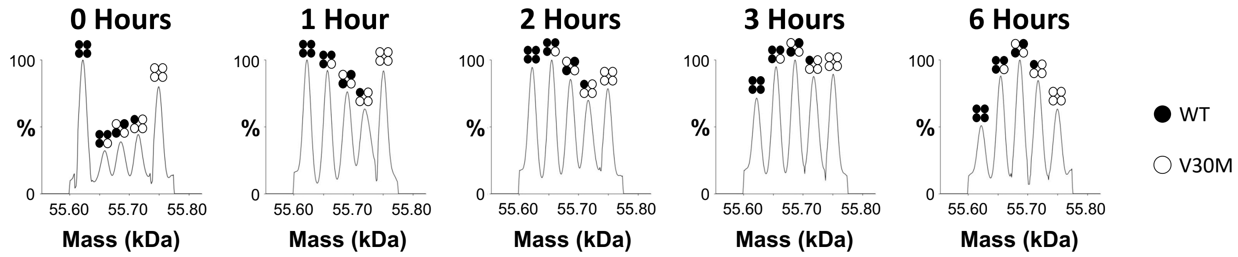

Figure S2: UniDec deconvoluted plots showing SUE between WT and V30M homotetramers in water at ambient temperature (21 °C). Notice that hTTR tetramers increase in abundance over time.

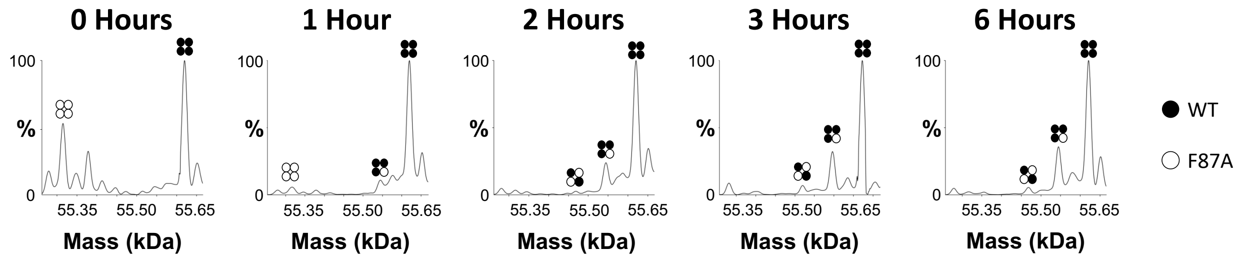

Figure S3: UniDec deconvoluted plots showing SUE between WT and F87A homotetramers in water at ambient temperature (21 °C). Notice that hTTR tetramers are not formed in great abundance because the F87A mutation destabilizes the tetramer complex.

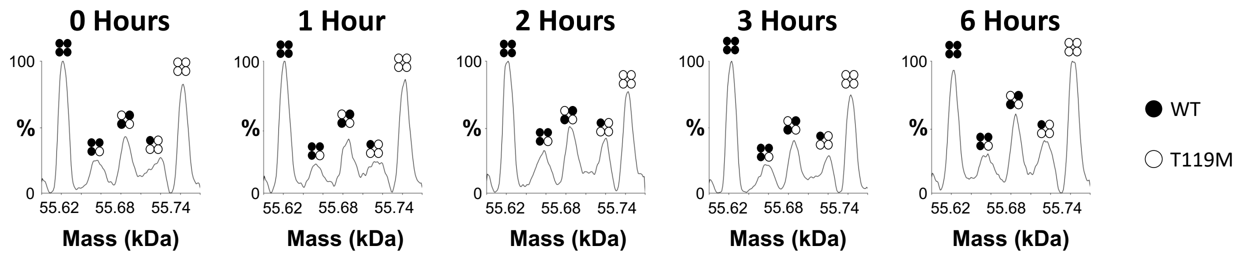

Figure S4: UniDec deconvoluted plots showing SUE between WT and T119M homotetramers in water at ambient temperature (21 °C). Notice that hTTR tetramers are not formed in great abundance because the T119M mutation stabilizes the homotetramer complex.

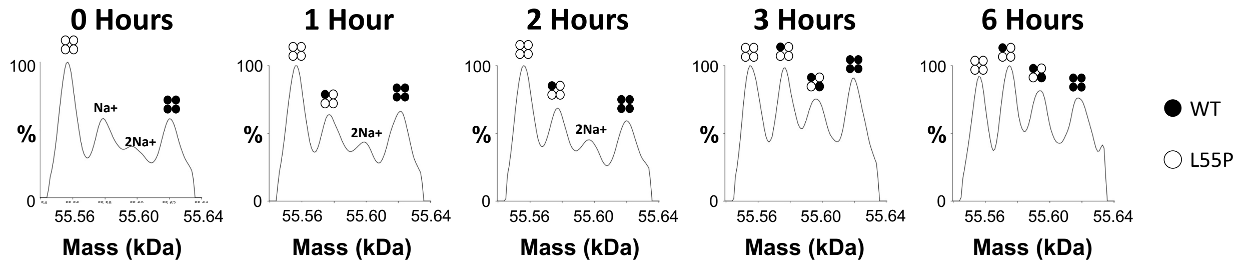

Figure S5: UniDec deconvoluted plots showing SUE of WT and L55P homotetramers in 20 mM ammonium acetate. Notice that hTTR tetramer abundances do not increase as readily in 20 mM ammonium acetate when compared to the tetramer abundances in water.

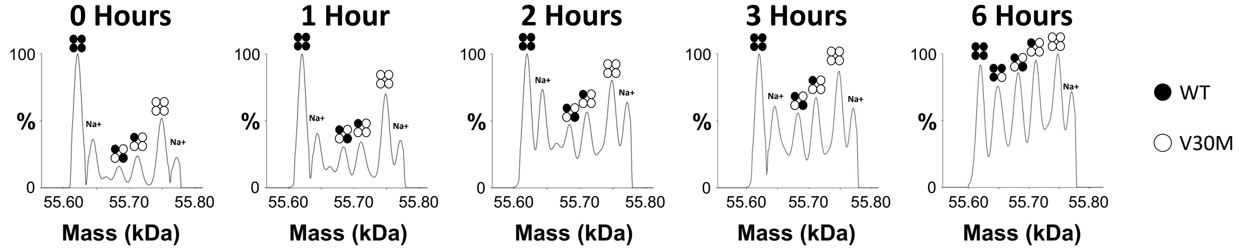

Figure S6: UniDec deconvoluted plots showing SUE of WT and V30M homotetramers in 20 mM ammonium acetate. Notice that hTTR tetramer abundances do not increase as readily in 20 mM ammonium acetate when compared to the tetramer abundances in water.

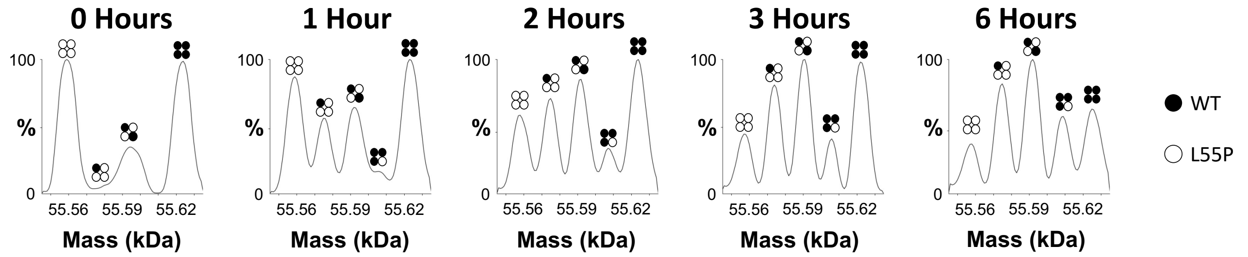

Figure S7: UniDec deconvoluted plots showing SUE between WT and L55P homotetramers in water at 35 °C. Notice that hTTR tetramers increase in abundance over time but not as readily compared to 21 °C.

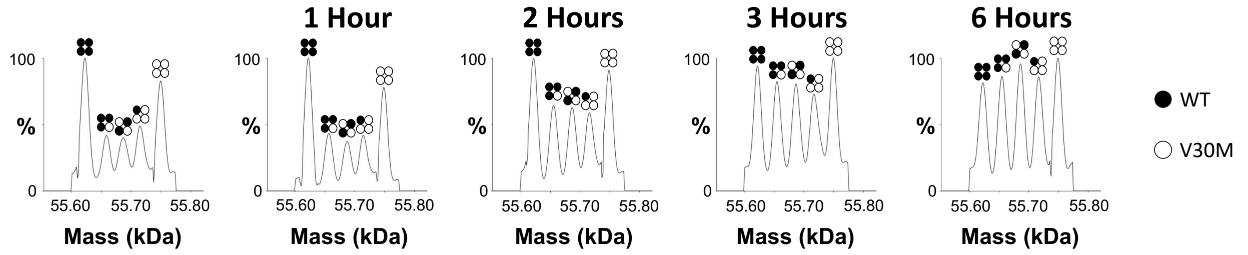

Figure S8: UniDec deconvoluted plots showing SUE between WT and V30M homotetramers in water at 35 °C. Notice that hTTR tetramers increase in abundance over time but not as readily compared to 21 °C.

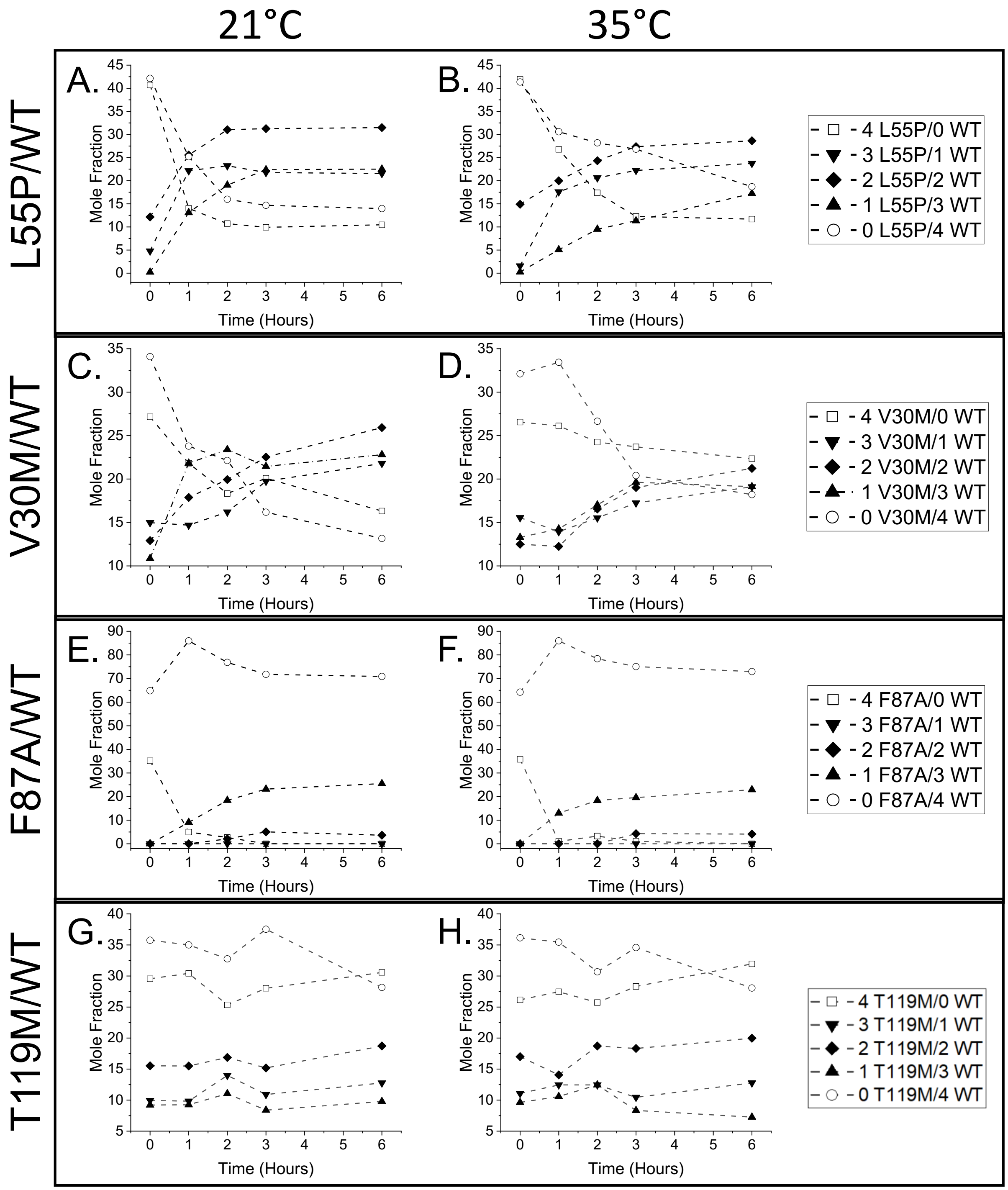

Figure S9: Mole fraction plots of homotetramers and hybrid tetramers in SUE solutions containing wt-TTR and L55P at A.) 21 °C and B.) 35 °C, V30M at C.) 21 °C and D.) 35 °C, F87A at E.) 21 °C and F.) 35 °C, and T119M at G.) 21 °C and H.) 35 °C. The lines connecting the datapoints are not intended to convey kinetic data.

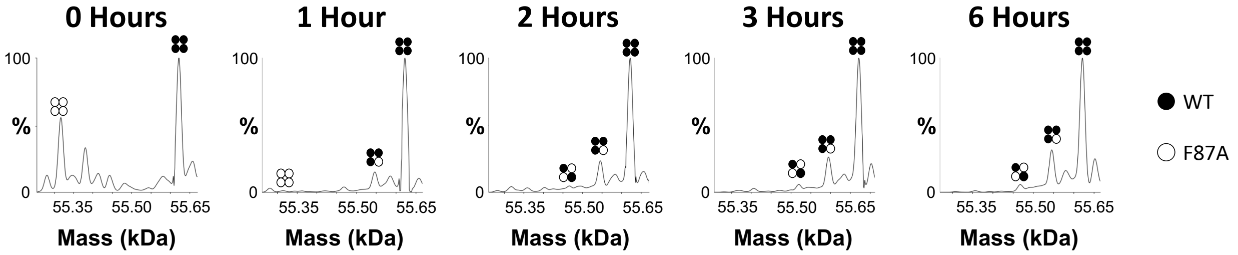

Figure S10: UniDec deconvoluted plots showing SUE between WT and F87A homotetramers in water at 35 °C. Notice few complexes containing F87A complexes are formed under these conditions.

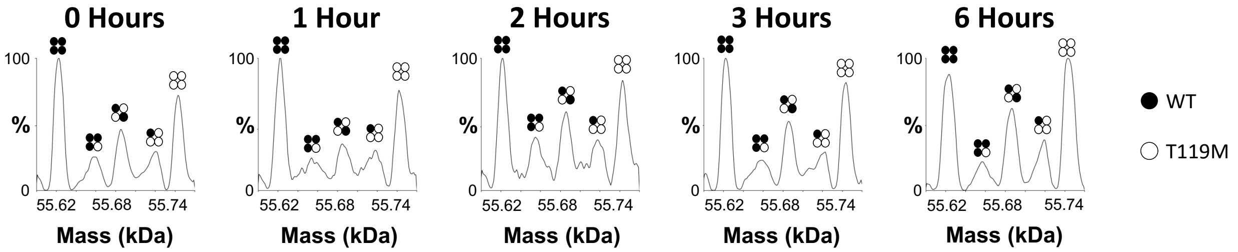

Figure S11: UniDec deconvoluted plots showing SUE between WT and T119M homotetramers in water at 35 °C. Notice that hTTR complexes are not made in abundance under these conditions.

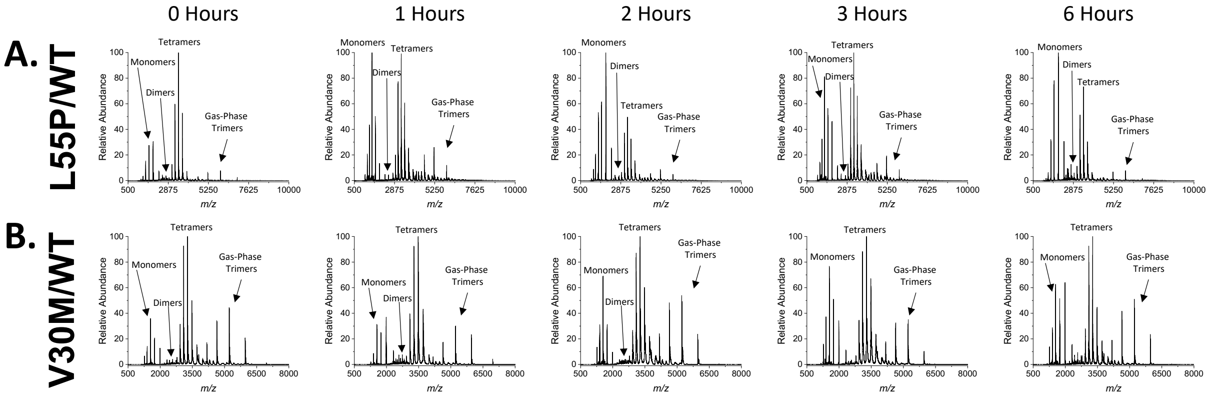

Figure S12: Raw spectra collected during SUE between A.) L55P and WT homotetramers and B.) V30M and WT homotetramers. Notice that monomers and dimers are observed in addition to tetramers, but that solution phase trimers are not present. The trimers observed are released by tetramers in the gas phase.

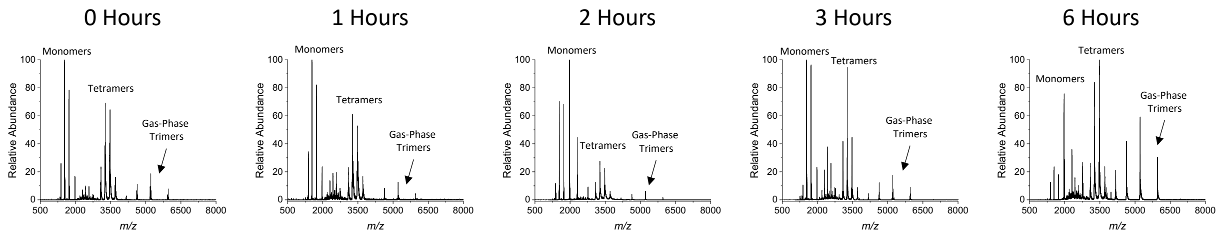

Figure S13: Raw spectra collected during SUE between F87A and WT homotetramers in water at 21 °C. The abundance of the monomers suggests that the F87A subunits do not readily form tetramer complexes.

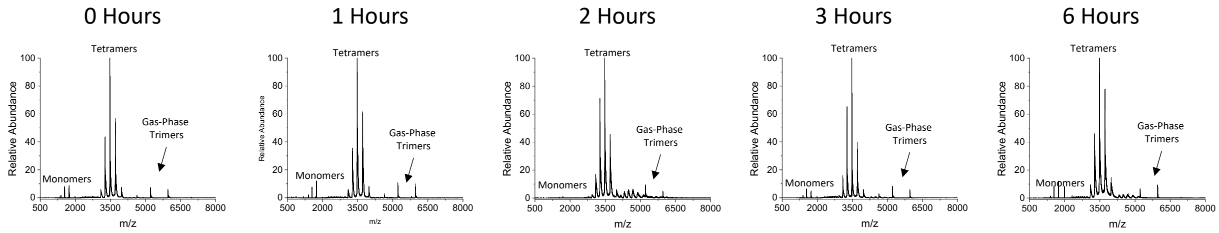

Figure S14: Raw spectra collected during SUE between T119M and WT homotetramers. The spectra show that monomers are not present in abundance suggesting that the T119M mutation stabilizes the tetramer complex.

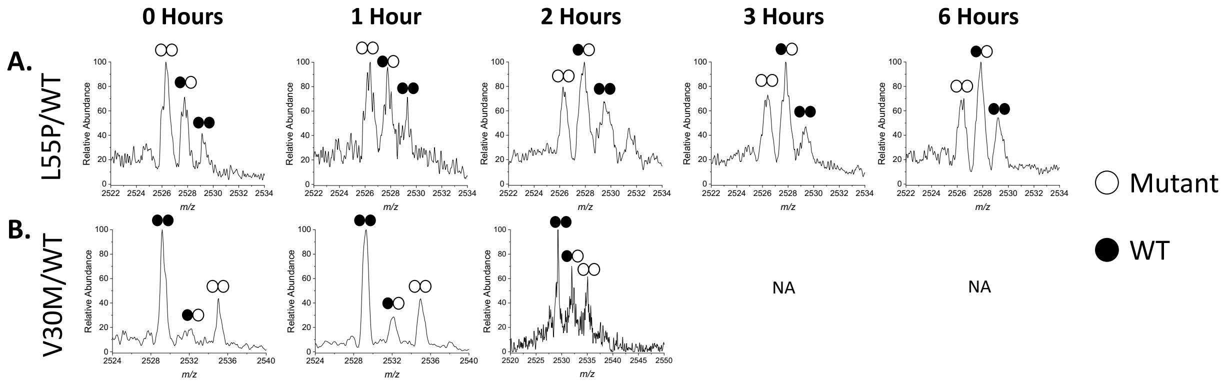

Figure S15: Raw spectra of the 11+ dimer charge state during SUE between A.) L55P and WT homotetramers and B.) V30M and WT homotetramers revealing that both homodimers and heterodimers are present at various time points.

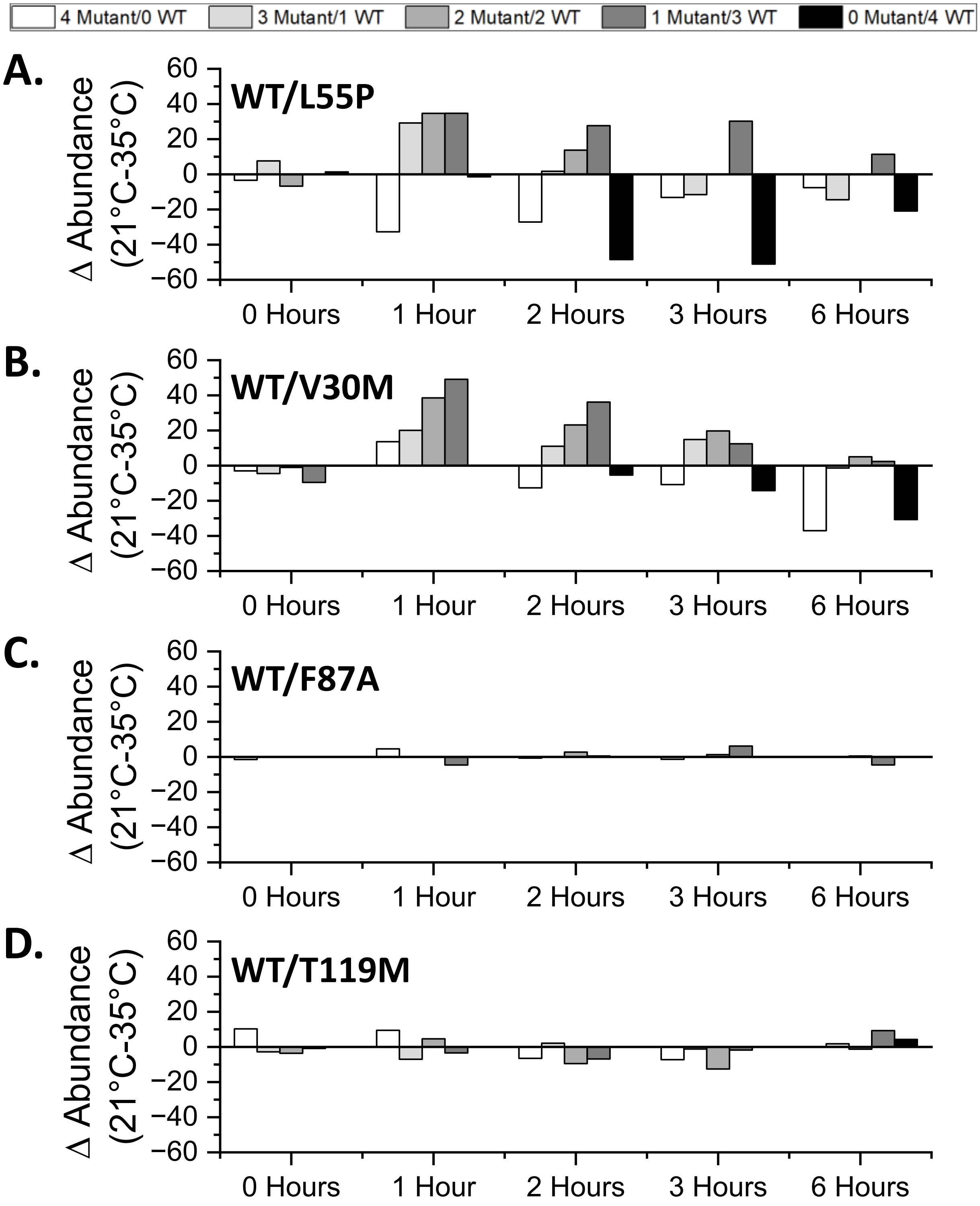

Figure S16: The difference in relative abundance for tetramer signals at 21 °C (Figure 1) and 35°C (Figure 2) for solutions initially containing wt-TTR and A.) L55P, B.) V30M, C.) F87A, and D.) T119M homotetramers. The data show that for L55P and V30M, temperature has a large effect on the products formed at early time points, whereas for F87A and T119M temperature does not affect the products formed.
